# Association between Knee Osteoarthritis, Cardiovascular Risk Factors, and the Framingham Risk Score in South Koreans: A Cross-Sectional Study

**DOI:** 10.1371/journal.pone.0165325

**Published:** 2016-10-20

**Authors:** Ho Sun Kim, Joon-Shik Shin, Jinho Lee, Yoon Jae Lee, Me-riong Kim, Young-Hyeon Bae, Ki Byung Park, Eun-Jung Lee, Joo-Hee Kim, In-Hyuk Ha

**Affiliations:** 1 Jaseng Spine and Joint Research Institute, Jaseng Medical Foundation, Seoul, Republic of Korea; 2 Department of Korean Rehabilitation Medicine, College of Korean Medicine, Dae-Jeon University, Daejeon, Republic of Korea; 3 Medical Research Division, Korea Institute of Oriental Medicine, Daejeon, Republic of Korea; University of Oslo, NORWAY

## Abstract

**Background:**

Osteoarthritis is a significant burden on personal health and for social cost, and its prevalence is rising. Recent research has revealed an association between osteoarthritis and cardiovascular disease, and this study uses the Framingham risk score (FRS), which is widely used as a composite index of cardiovascular risk factors, to investigate the association between osteoarthritis and various cardiovascular risk factors.

**Methods:**

A total 9,514 participants aged 50 years or older who received knee X-ray diagnosis of the 5th Korean National Health and Nutrition Examination Survey (total surveyees = 24,173) released by the Korean Centers for Disease Control and Prevention was included for analysis. Knee osteoarthritis patients were defined as participants with K-L grade ≥2 on knee X-ray regardless of knee pain. The association between major cardiovascular risk factors (blood pressure, diabetes, cholesterol, and smoking habits), FRS, and knee osteoarthritis was analyzed, adjusting for various covariates.

**Results:**

Prevalence of knee osteoarthritis in Koreans aged ≥50 years was 36.6%, and higher in women (men: 24.9%, women: 45.4%). Prevalence of knee osteoarthritis in participants with hypertension was significantly higher than those without hypertension (fully adjusted odds ratio (OR) 1.26; 95% confidence interval (CI) 1.08–1.48). Knee osteoarthritis prevalence was also higher in participants with impaired fasting glucose or diabetes than those without (age, sex adjusted OR 1.19; 95% CI 1.00–1.41). Also, OR values increased statistically significantly with FRS as a continuous variable (fully adjusted OR 1.007; 95% CI 1.00–1.01).

**Conclusions:**

Prevalence of knee osteoarthritis was associated with hypertension and diabetes, which are major cardiovascular risk factors, and the FRS. Further studies on FRS pertaining to its relationship with osteoarthritis are warranted.

## Introduction

Osteoarthritis (OA) is an important public health issue that significantly restricts daily activities and degrades quality of life through cartilage and disc degeneration and osteophyte formation in joints of the extremities and spine. It is of particular importance in the elderly as it holds higher prevalence and has been shown to raise incidence of complication and mortality in surgical treatment in this population [1,2]. OA incurs extensive structural abnormalities in cellular tissue of cartilage, subchondral bone, synovium, capsule and ligaments, and is characterized by pain upon movement and functional limitation [3]. Knee OA is one of the most common joint dysfunctions and types of OA and is a major cause of gait disturbance in the older population. In the U.S. alone, the number of OA patients is expected to reach 67 million by 2030 [4]. The 2008 Korean National Health and Nutrition Examination Survey (KNHANES) reports self-recognized OA prevalence at 15.1% in ages 50–59, 24.3% for 60–69, and 29.8% for ages 70 or older [5]. Accordingly, knee OA is not only burdensome for personal health but also a heavy social affliction.

The etiology of OA is idiopathic and related pathways are known to be various [6]. General risk factors for knee OA include age and obesity [7], and menopause and genetic variation have also been found to be major risk factors in knee OA pathology [8,9]. Following reports that larger number of OA-affected joints is related with lower survival rate, interest in the relationship between OA and cardiovascular risk factors to determine relevant mechanisms is increasing [10]. Previous studies on cardiovascular indicators related to OA report that OA in the hands is associated with above average serum cholesterol in women [11], and that substantially higher blood glucose level was observed in women with OA compared to that in healthy individuals [12]. A cohort study conducted in Finland asserted that OA in the finger joints and death by cardiovascular disease were associated [13], and the third National Health and Nutrition Examination Survey (NHANES) found that OA patients have more cardiovascular risk factors than those without OA [14]. In addition to cardiovascular indexes, such diseases as hypertension and Type 2 diabetes mellitus (T2DM) were commonly observed in elderly knee OA patients, with data showing that 55% of knee OA patients aged 65 or over had hypertension and 13%, T2DM [14].

The Framingham Heart Study has set the standard for cardiovascular disease risk factors through 50+ years of cohort studies spanning from before cardiovascular disease onset and thus identifying various common factors contributing to its occurrence [15]. The Framingham risk score developed through these observations gives the 10-year risk rate for cardiovascular diseases by assessing various influential factors (e.g. sex, age, systolic blood pressure (SBP), hypertension treatment, smoking history, diabetes mellitus (DM), total cholesterol, and high density lipoprotein (HDL) cholesterol) [16]. As the Framingham risk score considers for a wide range of cardiovascular disease-related factors, it can be useful tool for broad investigation of the association between cardiovascular risk factors and OA.

The authors have previously studied the correlation between the Framingham risk score and chronic low back pain [17]. Chronic low back pain showed a strong association with history of cardiovascular diseases which remained after adjustment for several confounding variables, while the Framingham risk score was not associated with chronic low back pain. It was suggested that relative insensitivity to pain despite structural pathology may denote hypoalgesic mechanism involvement in hypertension. Perhaps for this reason, knee pain and radiological data are often taken into consideration together in knee OA diagnosis. However, as inclusion of pain criteria may interfere with interpretation of cardiovascular diseases risk factors in radiological OA, this study chose to use Kellgren-Lawrence (K-L) grades based on objective diagnostic imaging (simple X-rays) and excluded subjective knee pain data in determining the relationship between cardiovascular risk factors and OA using the Framingham risk score in adults with knee OA aged 50 or over from the 5^th^ KNHANES (2010–2012) data.

## Materials and Methods

### Study population

The sample used in this cross-sectional study is the 5th KNHANES data managed and released by the Korean Centers for Disease Control and Prevention. This data are collected by the Korean government as basic data for examination and estimation of general health state, health care perception and usage, and food consumption and nutrition status of the South Korean population at national and regional (city and province) level, and for incorporation into public health care policy and policy evaluation. Of 31,596 potential surveyees, 24,173 (76.5%) participated in the examination and survey, and the subjects of this study were limited to the 9,514 participants aged 50 years or older with knee joint diagnosis data (with no upper age restriction).

### Osteoarthritis

Knee OA patients were defined as participants with K-L grade ≥2 on knee X-ray in the 5th KNHANES. Although controversy regarding its classification system continues, specialists have been in agreement that OA diagnosis should be made based on radiological features, and the World Health Organization has adopted the K-L scale as standard criteria [18]. The evaluation method covers various radiological indexes including joint space narrowing; osteophyte formation; presence of cysts within subchondral bone; and bone margin sclerosis.

### Framingham risk score

The Framingham risk score is calculated as follows: Participants are classified into one of 9 age groups, and total cholesterol, HDL cholesterol, systolic blood pressure (SBP), and diastolic blood pressure (DBP) are categorized into 5 subgroups, respectively, with each subgroup assigned a correlating risk score by sex. Participants are also divided into binary groups by current smoking and DM status, respectively. The final score is the total determined through this 6 step process [19].

### Cardiovascular risk factors

As medication intake for cardiovascular risk factors may be associated with knee OA, analysis was conducted considering for medication. Participants were accordingly classified into those who responded that they took hyperlipidemia, hypertension, or DM medicine 15 days or more per month; and those with lower or no intake in the survey. Blood pressure used the average of the 2^nd^ and 3^rd^ measurements in SBP and DBP following 3 measurements to minimize measurement error, and concentrations of total cholesterol, HDL cholesterol, low density lipoprotein (LDL) cholesterol and serum glucose were assessed after an 8-hour fasting period, and triglycerides were assessed after at least a 12-hour period to heighten test validity.

Hypertension, hyperlipidemia, and DM classification complied with KNHANES criteria which are as follows.

▶ Hypertension: SBP ≥140mmHg, DBP ≥90mmHg or with intake of hypertensive medicine; prehypertension: 120mmHg≤ SBP ≤139mmHg or 80mmHg≤ DBP ≤89mmHg
▶ Hypercholesterolemia: total cholesterol ≥240mg/dl or with intake of cholesterol medicine; hypertriglyceridemia: triglyceride ≥200mg/dl; hypo-HDL cholesterolemia: HDL cholesterol <40mg/dl; hyper-LDL cholesterolemia: LDL cholesterol ≥130mg/dl
▶ DM: blood glucose ≥126mg/dl, oral intake of DM medication or insulin injection, or physician diagnosis; impaired fasting glucose: 109mg/dl≤ blood glucose ≤125mg/dl (normal <109mg/dl)

The upper-half (≥148mmHg) of patients with SBP values of ≥140mmHg were compared with borderline hypertension (120-139mmHg) and normal groups (≤119mmHg). Similarly, the upper-half (≥93mmHg) of patients with DBP of ≥90mmHg were compared with borderline (80-89mmHg) and normal pressure groups (≤79mmHg). The upper-half (total cholesterol ≥256mg/dl, triglycerides ≥251mg/dl, and HDL cholesterol ≤35mg/dl) of patients with abnormal total cholesterol, triglycerides, and HDL cholesterol concentration were compared to those with normal range, respectively. For glucose, patients with abnormal and impaired fasting blood glucose and those with normal levels were compared. Smoking status was also categorized into 3 groups for analysis (current smokers, ex-smokers, and nonsmokers).

### Covariates

General, socioeconomic, and lifestyle characteristics of participants were included as covariates, covering such items as sex, age, education level, income level, occupation, body mass index (BMI), current smoking status, alcohol intake, exercise, and experience of stress or depressive symptoms. General and socioeconomic items were stratified as follows.

▶ Education level: Elementary school graduation or lower; middle school graduation; high school graduation; and college graduation or higher (total 4 groups)
▶ Income level: Quartiles according to monthly average household income adjusted for with equalization (monthly household income divided by household member number) (total 4 groups)
▶ Occupation: Professional, manager, or administrative worker; office worker; service or retail industry worker; skilled agriculture or fisheries worker; equipment or machinery operator; simple labor worker; and unemployed (modified from the occupation categories of the 6th revision of the Korean Standard Classification of Occupation to better reflect current Korean circumstances, total 7 groups)
▶ BMI (kg/m^2^): <18.5; 18.5–24.9; and ≤25 (as determined through physical measurements, total 3 groups)

Lifestyle-related characteristics were evaluated with smoking, alcohol intake, and regular exercise as follows.

▶ Smoking (total 3 groups)
  - Current smoker: Smokers who have smoked 5 packs of cigarettes or more over their lifetimes, and currently smoke
  - Ex-smoker: Past smokers who have smoked 5 packs of cigarettes or more over their lifetimes, and currently do not smoke
  - Nonsmoker: Individuals to whom the above current smoker and ex-smoker standards do not apply
▶ Alcohol consumption (total 2 groups)
  - Regular alcohol intake: Consumption of alcohol once a month or more
  - Nonregular alcohol intake: No alcohol consumption for the past year or consumption of alcohol less than once a month
▶ Regular exercise (total 2 groups)
  - Regular physical exercise: Three or more sessions/week of ≥20 minutes of intense physical exercise that is highly strenuous or requires labored breathing over the past week (jogging, hiking, high speed biking), 5 or more sessions/week of ≥30 minutes of moderate physical exercise that is slightly strenuous or requires slightly labored breathing over the past week (e.g. swimming at a slow pace, doubles tennis, volleyball), or 5 or more sessions/week of ≥30 minutes of walking over the past week
  - Nonregular physical exercise: Individuals to whom the above standards do not apply
▶ Mental health state: Experience of stress and depressive symptoms; experience of stress or depressive symptoms; no experience of stress or depressive symptoms (total 3 groups)

### Statistical analysis

Difference in participant characteristics by osteoarthritis prevalence (K-L grade≥2) was assessed using chi-square and independent t-test, and adjustment for covariates using logistic regression analysis was conducted to assess whether such factors as cardiovascular disease, the Framingham risk score, hypertension, hyperlipidemia, LDL-cholesterol, DM, medicine intake, serum lipid levels, blood glucose, and smoking were associated with OA. The Statistical Packages for Social Science program for Windows 11.0 (SPSS Inc., Chicago, IL, U.S.A.) was used. Population weights were applied to reflect the Korean population in adjusting for cluster sample distribution regarding primary extraction unit, stratification factors, and weighting.

### Ethics Statement

Interviewers were not informed about subjects prior to conducting interviews, and all participants gave written informed consent to participate. The protocol was approved by the Institutional Review Board (IRB) of Jaseng Hospital of Korean Medicine in Seoul, Korea (IRB approval number: JASENG 2016-04-003).

## Results

Women had higher prevalence of knee OA compared to men (men: 24.9%; women: 45.4%), and regarding income quartiles, all quartiles displayed a trend toward lower knee OA prevalence with higher income (lowest income quartile 39.5%; middle low quartile 37.6%; mid-upper quartile 35.2%; and highest quartile 33.8%, respectively). Contrary to our assumption that smoking would have a deleterious effect on OA as a risk factor of cardiovascular disease, statistical analysis showed that current smokers have lower knee OA prevalence than ex-smokers or nonsmokers, and that ex-smokers have lower prevalence compared to nonsmokers (42.9% in nonsmokers, 28.1% in ex-smokers, and 23.0% in current smokers, respectively, of the knee OA group). In addition, higher BMI was associated with increased knee OA prevalence, and individuals with hypertension, hyperlipidemia, or DM medicine intake had respectively higher knee OA prevalence compared to those with no intake (Table 1).

**Table 1:** Characteristics of Koreans aged 50 years or over participating in the 5th KNHANES.

Factor	Subgroup	Knee osteoarthritis prevalence^a^		
		No	Yes	p value
		(n = 6,031) (%)	(n = 3,483) (%)	
Age (years)	(mean±SD)	59.8±8.4	67.7±9.5	< .0001^c^
Sex	Male	3053 (75.1)	1012 (24.9)	< .0001^b^
	Female	2978 (54.7)	2471 (45.4)	
Household income	Low	1417 (60.5)	926 (39.5)	0.0308^b^
	Middle low	1464 (62.4)	882 (37.6)	
	Mid-upper	1537 (64.8)	836 (35.2)	
	High	1529 (66.2)	781 (33.8)	
Education	≤Elementary school	2245 (50.8)	2171 (49.2)	< .0001^b^
	Middle school	1089 (69.1)	486 (30.9)	
	High school	1625 (76.5)	500 (23.5)	
	≥College	884 (83.1)	180 (16.9)	
Occupation	Professional, manager, or administrative worker	425 (85.2)	74 (14.8)	< .0001^b^
	Office worker	201 (85.5)	34 (14.5)	
	Service or retail industry worker	681 (74.6)	232 (25.4)	
	Skilled agriculture or fisheries worker	744 (57.4)	553 (42.6)	
	Technician, equipment or machinery operator or manufacturer	578 (81.3)	133 (18.7)	
	Simple labor worker	658 (63.0)	387 (37.0)	
	Unemployed	2545 (57.0)	1923 (43.0)	
Current smoker	No	4738 (61.2)	3000 (38.8)	< .0001^b^
	Yes	1106 (77.0)	331 (23.0)	
Smoking status	Nonsmoker	3188 (57.1)	2395 (42.9)	< .0001^b^
	Ex-smoker	1550 (71.9)	605 (28.1)	
	Current smoker	1106 (77.0)	331 (23.0)	
Alcohol consumption	No	3023 (57.8)	2206 (42.2)	< .0001^b^
	Yes	2809 (71.6)	1115 (28.4)	
BMI (kg/m^2^)	<18.5	210 (75.5)	68 (24.5)	< .0001^b^
	<25	4040 (68.1)	1891 (31.9)	
	≥25	1770 (53.8)	1520 (46.2)	
Mental health state	No stress nor depressive symptoms	4164 (64.0)	2341 (36.0)	0.2497^b^
	Stress or depressive symptoms	1202 (63.6)	687 (36.4)	
	Stress and depressive symptoms	477 (61.6)	297 (38.4)	
Regular exercise	No	3074 (62.5)	1848 (37.6)	0.1140^b^
	Yes	2750 (65.2)	1466 (34.8)	
SBP (mmHg)	(mean±SD)	125±17.3	130±17.9	< .0001^c^
DBP (mmHg)	(mean±SD)	78.2±10.2	76.6±10.6	< .0001^c^
Total cholesterol (mg/dl)	(mean±SD)	194.9±37.1	195.8±37.7	0.3729^c^
HDL-cholesterol (mg/dl)	(mean±SD)	48.0±11.5	47.5±11.1	0.1977^c^
LDL-cholesterol (mg/dl)	(mean±SD)	118.0±33.9	120.5±33.1	0.2135^c^
Triglycerides (mg/dl)	(mean±SD)	150.4±115.6	143.5±100.4	0.0234^c^
Glucose (mg/dl)	(mean±SD)	102.9±25.3	104.0±25.0	0.1111^c^
FRS (%)	(mean±SD)	17.4±13.9	20.7±15.4	< .0001^c^
Hypertension medication	No	4010 (70.0)	1722 (30.0)	< .0001^b^
	Yes	1835 (53.1)	1623 (46.9)	
Hyperlipidemia medication	No	5214 (64.0)	2927 (36.0)	0.0069^b^
	Yes	628 (60.2)	415 (39.8)	
Diabetes oral medicine	No	5196 (64.6)	2844 (35.4)	< .0001^b^
	Yes	658 (56.7)	503 (43.3)	
Diabetes insulin injection	No	5775 (63.6)	3301 (36.4)	0.7671^b^
	Yes	79 (63.2)	46 (36.8)	
Physician diagnosis of diabetes	No	5083 (64.6)	2781 (35.4)	0.0003^b^
	Yes	775 (57.6)	570 (42.4)	

^a^ Knee osteoarthritis: K-L grade ≥2

^b^ p value for Rao-scott chi-square test

^c^ p value for t-test

KNHANES: Korean National Health and Nutrition Examination Survey; BMI: Body mass index; SBP: Systolic blood pressure; DBP: Diastolic blood pressure; HDL: High density lipoprotein; LDL: Low density lipoprotein; FRS: Framingham risk score

Hypertensives displayed a statistically significantly higher prevalence of knee OA than nonhypertensives (fully adjusted odds ratio (OR) 1.26; age and sex adjusted OR 1.48). Individuals with impaired fasting glucose or diabetes also showed statistically significantly higher knee OA prevalence compared to those that did not (age and sex adjusted OR in impaired fasting glucose 1.16; age and sex adjusted OR in diabetes 1.19) (Table 2).

**Table 2:** Association between cardiovascular risk factors and knee osteoarthritis prevalence^a^.

Factors		n (case^b^)	Crude			Adjusted for age and sex			Fully adjusted^c^		
			OR	95% CI	p	OR	95% CI	p	OR	95% CI	p
Hypertension	Normal	2189 (559)									
	Prehypertension	2263 (706)	1.12	0.96–1.31	0.1386	1.07	0.90–1.26	0.4367	1.00	0.84–1.19	0.9766
	Hypertension	4729 (2077)	2.13	1.85–2.46	< .0001^d^	1.48	1.27–1.72	< .0001^d^	1.26	1.08–1.48	0.0035^d^
Hypercholesterolemia	No	6584 (2279)									
	Yes	1865 (706)	1.20	1.06–1.35	0.0033^d^	1.06	0.93–1.21	0.3697	0.99	0.86–1.14	0.8783
Hypertriglyceridemia	No	6271 (2314)									
	Yes	1318 (451)	0.84	0.73–0.98	0.0289^d^	1.04	0.88–1.23	0.658	0.91	0.76–1.08	0.2881
Hypoalphalipoproteinemia	No	6358 (2236)									
	Yes	2307 (836)	1.00	0.88–1.15	0.9891	1.05	0.91–1.22	0.5116	0.95	0.81–1.12	0.545
Hyper-LDL cholesterolemia	No	1293 (352)									
	Yes	737 (242)	1.18	0.93–1.49	0.1723	1.20	0.94–1.54	0.1382	1.13	0.86–1.47	0.3831
Glucose	Normal	4916 (1654)									
	Impaired fasting glucose	2081 (738)	1.08	0.95–1.24	0.2479	1.16	1.00–1.34	0.0496^d^	1.02	0.87–1.19	0.8201
	Diabetes	1452 (593)	1.33	1.14–1.54	0.0002^d^	1.19	1.00–1.41	0.0446^d^	1.06	0.89–1.27	0.5155

^a^ Knee osteoarthritis: K-L grade ≥2

^b^ Number of cases indicates the number of cases with K-L grade 2–4

^c^ Adjusted for age, sex, education level, household income, occupation, BMI, present smoking status, alcohol consumption, regular exercise habits and mental health

^d^ p < 0.05

OR: Odds ratio; CI: Confidence interval; LDL: Low density lipoprotein

Analyses of individuals with no medication for hyperlipidemia, hypertension, or DM (n = 5056) resulted in nonsignificant estimates by SBP, DBP, total cholesterol, HDL cholesterol, triglycerides, glucose, and current smoking status (Table 3).

**Table 3:** Knee osteoarthritis prevalence in individuals with no cardiovascular related conventional medicine intake^a^^,^^b^.

Factors	Subgroup	Total	Crude			Adjusted for age and sex			Fully adjusted		
		n (case)	OR	95% CI	p	OR	95% CI	p	OR	95% CI	p
SBP (mmHg)^c^	≥148	493 (197)									
	<148	409 (149)	1.00	0.71–1.41	0.985	1.12	0.77–1.63	0.5595	1.059	0.73–1.54	0.7616
	<140	1945 (620)	0.77	0.61–0.99	0.0404^e^	0.94	0.71–1.26	0.6874	0.939	0.71–1.25	0.6653
	<120	2209 (535)	0.56	0.44–0.72	< .0001^e^	0.77	0.58–1.03	0.0805	0.84	0.64–1.11	0.2172
DBP (mmHg)^c^	≥93	350 (90)									
	<93	308 (101)	1.66	1.13–2.46	0.0106^e^	1.38	0.9–2.1	0.1379	1.393	0.9–2.16	0.139
	<90	1488 (426)	1.17	0.85–1.6	0.34	0.84	0.59–1.2	0.3369	0.848	0.59–1.23	0.3838
	<80	2910 (884)	1.45	1.08–1.95	0.0123^e^	0.79	0.56–1.11	0.1654	0.872	0.61–1.24	0.4437
Hypercholesterolemia (mg/dl)^c^	≥256	314 (105)									
	<256	303 (96)	1.02	0.67–1.56	0.9119	0.92	0.59–1.46	0.7346	1.007	0.62–1.64	0.9759
	<240	2096 (587)	0.86	0.63–1.17	0.3388	0.86	0.61–1.22	0.4022	0.972	0.67–1.42	0.882
	<193	2012 (572)	0.87	0.63–1.2	0.3846	0.87	0.61–1.24	0.4397	1.053	0.71–1.56	0.796
Hypertriglyceridemia (mg/dl)^c^	≥251	333 (98)									
	<251	331 (93)	1.11	0.74–1.67	0.6233	0.84	0.53–1.31	0.4312	0.926	0.59–1.46	0.742
	<200	1808 (544)	1.21	0.88–1.65	0.2394	0.84	0.59–1.19	0.3255	0.994	0.7–1.42	0.9752
	<103	1784 (543)	1.37	1.01–1.86	0.0465^e^	0.95	0.68–1.34	0.7743	1.288	0.91–1.82	0.1547
Hypoalphalipoproteinemia (mg/dl)^c^	<36	535 (161)									
	<40	600 (181)	1.08	0.77–1.53	0.6534	1.11	0.76–1.61	0.5978	1.046	0.71–1.55	0.8223
	<50	1794 (549)	1.06	0.8–1.4	0.688	1.06	0.79–1.43	0.6933	1.155	0.84–1.59	0.38
	≥50	2004 (553)	0.95	0.72–1.25	0.7015	0.92	0.68–1.24	0.573	1.077	0.78–1.49	0.6504
Hyper-LDL cholesterolemia (mg/dl)^c^	≥148	245 (68)									
	<148	239 (70)	0.96	0.6–1.54	0.8616	0.99	0.6–1.62	0.9652	1.003	0.6–1.68	0.9919
	<130	346 (76)	0.78	0.5–1.2	0.2607	0.84	0.53–1.35	0.4824	0.898	0.53–1.51	0.6868
	<106	337 (71)	0.70	0.45–1.08	0.1083	0.72	0.45–1.14	0.1614	0.885	0.53–1.48	0.641
Glucose (mg/dl)^c^	≥126	198 (64)									
	<126	361 (114)	0.83	0.54–1.27	0.3814	0.83	0.51–1.36	0.4606	0.762	0.44–1.33	0.3376
	<110	4363 (1263)	0.80	0.56–1.15	0.2311	0.67	0.44–1.02	0.0599	0.717	0.45–1.15	0.1665
Smoking status^d^	Current smokers	897 (185)									
	Ex-smokers	1128 (271)	1.20	0.93–1.56	0.1676	0.96	0.72–1.28	0.7625	0.891	0.66–1.2	0.4433
	Nonsmokers	3039 (1044)	2.23	1.8–2.76	< .0001^e^	1.31	0.98–1.75	0.0671	1.197	0.88–1.62	0.2486

^a^ Knee osteoarthritis: K-L grade ≥2

^b^ Subjects without intake of medication for hyperlipemia, hypertension or diabetes

^c^ Adjusted for age, sex, education level, household income, occupation, BMI, present smoking status, alcohol consumption, regular exercise habits and mental health in fully adjusted model

^d^ Adjusted for age, sex, education level, household income, occupation, BMI, alcohol consumption, regular exercise habits and mental health in fully adjusted model

^e^ p < 0.05

OR: Odds ratio; CI: Confidence interval; SBP: Systolic blood pressure; DBP: Diastolic blood pressure; LDL: Low density lipoprotein

In analysis of the Framingham risk score as a continuous variable, OR increase with the Framingham risk score was statistically significant (fully adjusted OR 1.007, p = 0.0424). Knee OA prevalence also displayed a tendency to increase with categorical Framingham risk scores divided into quartiles of <8; 8≤ to <15, 15≤ to <26, and ≥26 (age and sex adjusted trend, p = 0.0005; fully adjusted trend, p = 0.0763) (Table 4).

**Table 4:** Association between the Framingham risk score and knee osteoarthritis prevalence^a^.

Factors				Crude			Adjusted for age and sex			Fully adjusted		
			n (case)	OR	95% CI	p	OR	95% CI	p	OR	95% CI	p
FRS	Continuous variable			1.015	1.01–1.02	< .0001	1.009	1.00–1.02	0.0031	1.007	1.00–1.01	0.0424
FRS	Categorical variable	Trend		1.227	1.17–1.29	< .0001	1.159	1.07–1.26	0.0005	1.081	0.99–1.18	0.0763
		q1 (<8)	2127 (527)									
		q2 (8≤ <15)	2058 (759)	1.428	1.20–1.70	< .0001	1.338	1.10–1.62	0.0032	1.163	0.95–1.42	0.1424
		q3 (15≤ <26)	2105 (847)	1.747	1.48–2.06	< .0001	1.546	1.25–1.91	< .0001	1.264	1.02–1.57	0.035
		q4 (≥26)	2105 (830)	1.864	1.57–2.21	< .0001	1.557	1.20–2.02	0.0009	1.255	0.96–1.65	0.1029

^a^ Comparison between K-L grade 0–1 and 2–4 groups

OR: Odds ratio; CI: Confidence interval; FRS: Framingham risk score

## Discussion

Analysis of data from the 5^th^ KNHANES shows that prevalence of knee OA was higher in those with hypertension than in those with normal blood pressure, and that it was higher in those with impaired fasting glucose or diabetes than in those without. Moreover, OR values for knee OA increased with higher Framingham risk scores.

Monson RR et al. discovered that mortality was higher in arthritic patients [20], and the number of investigations on the association between cardiovascular risk factors and arthritis is increasing in an attempt to identify how joint disease influences mortality. High prevalence of vascular disorders [21,22] and cardiovascular risk factors [14] have been reported in individuals with OA, and a positive correlation between OA and hypercholesterolemia has been demonstrated [23]. More recently, osteoarthritic change in the hand joints was found to be related to aortic calcification [24]. Correlations may also be drawn between intervertebral disc and articular cartilage, and research has shown that lumbar atherosclerotic calcification on CT scans was related to the degree of disc degeneration [25]. A 3 year follow-up study on the incidence and progression of knee OA reported a strong association between hypertension and impaired glucose tolerance with knee OA occurrence [26]. Several studies report that the correlation between hypertension and knee OA persists after adjustment for overweight [27,28], and a 2013 study relates how knee OA patients with hypertension and T2DM displayed greater bone loss in the subchondral plate than those without [6].

A 2015 review article on the association between OA and cardiovascular disease gives a point-by-point summary of the basic mechanical, causal and shared risk factors potentially explaining the relationship: osteoarthritis and cardiovascular disease may concur in a large patient proportion from common risk factors (ageing, obesity and gender), the interrelationship between OA and cardiovascular disease (physical inactivity in OA such as walking disability resulting in cardiovascular disease, high intake of analgesics), and common etiology (especially low-grade chronic inflammation and certain molecular pathways) [29]. Various similarities in the etiology of OA and cardiovascular diseases have been found, supporting the claim that the association accounted in numerous cohort and epidemiological studies is not accidental. Z39Ig, a transmembrane protein existing in human carotid arterial plaque, was also found in osteoarthritic synovial lining [30], and this discovery has led to wider acceptance of the pathological link between OA and cardiovascular disease. Subchondral bone, which is highly vascularized, has been drawing interest as a potential factor in OA pathogenesis, and the subchondral vascular proliferation into degenerate articular cartilage and ensuing venous engorgement is closely related to the cartilage degeneration and osteophyte growth seen in OA [31]. Taking into account that subchondral bone is usually the first structure to be affected, followed by cartilage degeneration, it has been put forth that subchondral vascular pathology accelerates OA progression through nutritional interference [32], hypervascularity weakening bone structure, and alternately, ischemia leading to necrotic bone in advanced stages [31]. While the meniscus is avascular, adjacent tissues are vascularized. The superior, middle, and inferior genicular branches of the popliteal artery form the parameniscal capillary plexus in the synovial tissue of the marginal meniscus and articular capsule and supplies blood to 10–30% of the medial meniscus width and 10–25% of the lateral meniscus. Therefore, association with cardiovascular diseases may be higher in knee OA compared to other OA regions.

Hypertension and hyperglycemia showed strong correlations with OA in this study. Most previous literature favors the concept of ischemia in etiology and pathogenesis of OA [31], and a 2007 study on the association between vascular pathology and the onset or development of OA [33] purported how decreased peripheral blood flow associated with hypertension may induce subchondral ischemia impeding nutrition and gas exchange in articular cartilage and resulting in osteocyte cell death in subchondral bone. Moreover, OA chondrocytes exposed to hyperglycemia were shown to be unable to downregulate glucose transporter-1 (GLUT-1) [34]. It can therefore be purported that dysfunctional GLUT-1 downregulation may be part of the pathologic mechanism contributing to high glucose and further degeneration of chondrocytes, thus accelerating the progression of OA. Also, while controversy continues concerning the association or lack thereof between smoking and OA occurrence, the results of this study displayed partially significant results in crude values. However, significance was not maintained in adjusted models, and this nonsignificance is in concordance with results from a previous study for smoking behavior including direct smoking and knee OA prevalence and total (knee and hip) OA prevalence using the same 5th KNHANES dataset [35].

Some strengths of this study include that it employs radiological imaging in analysis of a large patient data sample representing the South Korean population, and that the health survey, examination, and radiology readings were performed systematically by trained experts, whereas several previous studies are limited in that they use hospital data in a limited patient pool and are consequently more susceptible to selection bias. An additional strength of this study is that it used the Framingham risk score for more inclusive analysis of the correlation between cardiovascular risk factors and OA, adjusting for various potential confounding variables (age, sex, BMI, income level, education level, occupation, smoking, drinking, and physical exercise). Also, this study used results obtained after an 8-hour (excluding triglyceride) and 12-hour (triglyceride) fasting period in measurement of cholesterol and triglyceride to secure test validity. We would also like to draw attention to the fact that subjects were not simply divided into tertiles in analysis of risk factors, but that we used the upper half of the abnormal range as Asians are generally healthier than Western populations regarding cardiovascular risk factors. However, this study shares the inherent limitation of cross-sectional studies lacking follow up in being unable to determine causal relationship and internal validity, and the possibility of sampling error as a survey study. Some inconsistencies exist between subgroup categories in comparisons with normal range in this study. For example, while hypertension and diabetes comparisons are trichotomous (e.g. normal, prehypertension, hypertension), those for lipid related factors are dichotomous (e.g. normal, hypercholesterolemia). The reason for this discrepancy in criteria was not by intentional design but an inherent limitation arising from retrospective use of raw data collected by third parties.

Another major limitation of this study is the small effect size of results. The p-value of impaired fasting glucose and diabetes in the age and sex adjusted model is close to 0.05 and nonsignificant in the fully adjusted model. Also, regarding Framingham risk scores, if we consider the difference in mean Framingham risk score between those with and without knee OA, there is a difference of about 3 points based on the estimated OR which would correspond to an approximately 2% increased odds of knee OA. As the Framingham risk score is the composite score of various cardiovascular risk factors including hypertension which displayed strong positive associations with knee OA prevalence and other factors of both positive and negative, significant and nonsignificant relations, the overall effect would seem to have been offset. Still, these results show that OA prevalence is slightly higher in the diabetic group compared to the normal group and that the continuous Framingham risk score also presents an effect—albeit fairly small. However, as these outcomes may be due to chance, they warrant careful interpretation.

The reason for further analysis in individuals with no cardiovascular related conventional medicine intake in determining the association between cardiovascular risk factors and OA was as regular intake of cardiovascular medicine may additionally affect the association between such risk factors and OA. Meanwhile, the group with no cardiovascular related medicine intake covers a wide spectrum ranging from healthy individuals to asymptomatic cardiovascular patients, patients of recent onset and patients actively choosing not to take medicine. The fact that OA prevalence in individuals with no cardiovascular related medicine intake did not reach statistical significance is worth note and may be interpreted from multiple perspectives. For instance, this may be due to the difference in sampling size compared to the total population; or that while total analysis (inclusive of individuals with cardiovascular related medicine intake) includes chronic hypertension or diabetes patients whose conditions failed to be controlled by regular medicine intake, analysis of individuals with no cardiovascular related medicine intake only includes hypertension or diabetes patients of more recent onset or with transitory symptoms. Future studies should bear in mind these limitations and refrain from overly broad interpretations.

In conclusion, knee OA was shown to be associated with such cardiovascular risk factors as hypertension and DM, and with the Framingham risk score, which is the composite total of various major cardiovascular factors, and these results may be due to the detrimental effect that atherosclerosis has on subchondral bone. Knee OA entails prolonged suffering in individuals along with substantial socioeconomic costs, and assessment of risk factors and comprehensive risk scores may help contribute to its prediction and clinical management. To the best of our knowledge, this is the first study investigating OA in relation to the Framingham risk score considering that previous research generally focuses on individual cardiovascular factors, and further longitudinal studies elucidating the causal relationship between the Framingham risk score and arthritis are warranted.
